# Fidelity Index Determination of DNA Methyltransferases

**DOI:** 10.1371/journal.pone.0063866

**Published:** 2013-05-06

**Authors:** Janine G. Borgaro, Nicole Benner, Zhenyu Zhu

**Affiliations:** New England Biolabs Inc., Ipswich, Massachusetts, United States of America; Institute of Enzymology of the Hungarian Academy of Science, Hungary

## Abstract

DNA methylation is the most frequent form of epigenetic modification in the cell, which involves gene regulation in eukaryotes and protection against restriction enzymes in prokaryotes. Even though many methyltransferases exclusively modify their cognate sites, there have been reports of those that exhibit promiscuity. Previous experimental approaches used to characterize these methyltransferases do not provide the exact concentration at which off-target methylation occurs. Here, we present the first reported fidelity index (FI) for a number of DNA methyltransferases. We define the FI as the ratio of the highest amount of methyltransferase that exhibits no star activity (off-target effects) to the lowest amount that exhibits complete modification of the cognate site. Of the methyltransferases assayed, M.MspI and M.AluI exhibited the highest fidelity of ≥250 and ≥500, respectively, and do not show star activity even at very high concentrations. In contrast, M.HaeIII, M.EcoKDam and M.BamHI have the lowest fidelity of 4, 4 and 2, respectively, and exhibit star activity at concentrations close to complete methylation of the cognate site. The fidelity indexes provide vital information on the usage of methyltransferases and are especially important in applications where site specific methylation is required.

## Introduction

In prokaryotes, DNA methyltransferases are part of restriction-modification systems, where both the methyltransferase and cognate restriction enzyme recognize the same DNA sequence. Methylated DNA is protected against cleavage from the paired restriction enzyme, whose purpose is to defend the host from foreign invaders [1]. Methyltransferases are also involved in chromosomal replication, gene regulation and DNA mismatch repair [2]. In eukaryotes, methyltransferases are involved in epigenetic control and gene expression [23].

In bacteria and archaea, DNA methyltransferases can be separated into three distinct classes depending on the location of the modification and type of reaction they catalyze. N6-methyladenine (m6A) and N4-methylcytosine (m4C) result from methylation of the amino moiety of adenine and cytosine, respectively, while 5-methylcytosine (m5C) is the result of methylation at the C5 position of cytosine. To modify DNA, the methyltransferases use S-adenosyl-L-methionine (SAM) as the donor of a methyl group [4], [5]. In eukaryotes, methyltransferases are in the m5C class and are responsible for CpG methylation, a key component in genomic imprinting and female X-inactivation [6], [7].

Many restriction enzymes exhibit star activity, an event in which cleavage occurs at a site that is one or more bases different from the recognition sequence [8]. In contrast, many of the methyltransferases that have been assayed for site specificity displayed exclusivity toward their cognate sites [9]. However, there have been reports of methyltransferases that promiscuously methylate sites close to their cognate sites [9], [10], [11], [12], [13], [14]. Among the first reported was M.EcoRI, which was shown to only require a duplex of AT base pairs for methylation. More recently, through single-molecule real-time (SMRT) DNA sequencing, which can directly detect methylated DNA bases, over-expressed M.EcoKDam from a high copy vector was shown to methylate sites differing from the target sequence by only one base [9], [15].

It has previously been shown that the over-expression of certain methyltransferases results in methylation at non-canonical sites [16]. However, the experimental approaches used to characterize these methyltransferases do not provide the exact concentration at which the off-target methylation (star activity) occurs. Here we present a method to determine the fidelity of DNA methyltransferases which can serve as experimental guidelines for methyltransferase usage.

## Materials and Methods

### Escherichia coli strains

C2566: [NEB *dam+/dcm*-] *fhuA2 lacZ::T7 gene1 [lon] ompT gal sulA11 R(mcr-73::miniTn10-*Tet^S^ *)2 [dcm] R(zgb-210::Tn10–*Tet^S^ *) endA1* Δ*(mcrC-mrr)114::IS10.*

ER2796 ( = DB24): *fhuA2* Δ *(lacZ)r1 glnV44 trp-31 dcm-6 his-1 zed-501::Tn10 argG6 rpsL104 dam-16::Kan xyl-7 mtl-2 metR1 mcr-62* Δ *(mcrB-hsd-mrr)114*.

### Cloning, expression and purification of methyltransferases

The genes for all methyltransferases were cloned into pTXB1 (NEB #N6707) and transformed into *E. coli* strain T7 Express (NEB #C2566). After selection on solid LB media containing ampicillin (100 µg/mL), individual colonies were used to inoculate 1 L of LB media containing ampicillin (100 µg/mL) and grown at 37°C to late log phase. Protein expression was then induced with 0.5 mM IPTG. After incubating overnight at 16°C, cells were harvested by centrifugation, resuspended in 25 mL of 10 mM Tris-HCl, 500 mM NaCl buffer, pH 8.0 (Buffer A) and sonicated at 4°C. Cell debris was removed by centrifugation and the cell free extract was then loaded onto a chitin column (NEB #S6651), pre-equilibrated with Buffer A. The column was then washed with 10 column volumes of Buffer A. For intein cleavage, 50 mL of Buffer A containing 30 mM dithiothreitol (DTT) was added to the column and incubated at 4°C overnight. Fractions containing the protein were eluted from the column and protein purity was analyzed by SDS**-**PAGE.

### Restriction enzyme digestion protection assay

Typically, a concentrated methyltransferase stock was first subjected to a series of 2-fold dilutions in diluent A (50 mM KCl, 10 mM Tris-HCl, 0.1 mM EDTA, 1 mM DTT, 200 µg/mL BSA, 50% glycerol (v/v), pH 7.4), resulting in 20 different concentrations (1x, 0.5x, 0.25x, etc.). A standard methylation assay consisted of a 30 µL reaction containing a fixed concentration of [Methyl^-3^H]-*S*-Adenosylmethionine (SAM, Perkin Elmer #NET155V001MC), 1 µg of N^6^-methyladenine free λ DNA (NEB #N3013), and varying concentrations of methyltransferase from the two-fold dilution series (making up 10% of the final volume) in the appropriate methyltransferase buffer (see below). The reaction mixture was incubated for 1 hour at 37°C. Following incubation, the DNA was purified with QIAquick columns (Qiagen #27106) and 100 ng of the newly purified methylated DNA was subjected to digestion by the restriction endonuclease (RE) that pairs with the methylase. For example, to determine the activity of M.HhaI, 100 ng of M.HhaI methylated DNA was subjected to 20 units of HhaI RE in NEB buffer 4 (50 mM potassium acetate, 20 mM Tris-acetate, 10 mM magnesium acetate, 1 mM DTT, pH 7.9) for 1 hour at 37°C. The reaction was then analyzed on a 0.8% agarose gel.

The reaction buffers used in each methyltransferase assay were as follows: For M.HhaI, M.AluI, M.EcoKDam, M.HpaII, and M.BamHI: 50 mM Tris-HCl, 5 mM 2-mercaptoethanol, 10 mM EDTA, pH 7.5; M.EcoRI: 50 mM Tris-HCl, 50 mM NaCl, 10 mM EDTA, pH 8.0; M.HaeIII: 50 mM Tris-HCl, 50 mM NaCl, 10 mM EDTA, pH 8.5; M.MspI: 50 mM Tris-HCl, 100 mM NaCl, 5 mM 2-mercaptoethanol, 10 mM EDTA, pH 7.5.

### Radioactive DNA methyltransferase assay

Analogous to the protection assay, a concentrated methyltransferase stock was first subjected to a series of 2-fold dilutions in diluent A. A radioactive methylation assay consisted of a 200 µL reaction containing a fixed concentration of SAM, 1 µg of *E. coli* genomic DNA (NEB #ER2796) pre-sheared to 200 bp fragments by a Covaris s-series sonicator (Covaris, MA), and varying concentrations of methyltransferase from the two-fold dilution series (making up 10% of the final volume) in the appropriate methyltransferase buffer (see above). The reaction mixture was incubated for 1 hour at 37°C. The reactions were stopped by flash freezing in an ethanol/dry ice bath. The samples were processed by applying the thawed reaction to a 2.5 cm DE81 membrane (GE Healthcare #3658–325) under air pressure using a vacuum manifold (Millipore, MA). The reaction was washed 3 times with 0.2 M ammonium bicarbonate, 3 times with deionized water and lastly, 3 times with ethanol. The membranes were dried and the amount of tritium incorporation was determined by standard scintillation counting for 1 min (Perkin Elmer TriCarb 2900TR). All reactions were performed in duplicate.

Additionally, we used M.HhaI as our model enzyme to determine if factors such as time, and glycerol, DNA and enzyme concentrations would influence star activity. FI incubation times were extended to 16 hours, glycerol concentrations were increased from a 5% final concentration to a 10% final concentration, DNA concentrations were halved from 1 µg to 500 ng and lastly, the amount of enzyme was increased by a factor of 4.

### Digestion of cognate sites with restriction enzyme

The digestion reaction contained 23 µg of *E. coli* genomic DNA pre-sheared to 1 kB fragments by a Covaris s-series sonicator, and 200 units of either MboI (NEB #R0147), HhaI (NEB #R0139) or AluI (NEB #R0137) restriction enzyme in NEB4. The reaction was incubated for 1 hour at 37°C. Following incubation, the DNA was purified with QIAquick columns and subsequently used as a substrate in the radioactive methylation assay (see above).

## Results

### Purification of DNA methyltransferases

**Figure 1:**
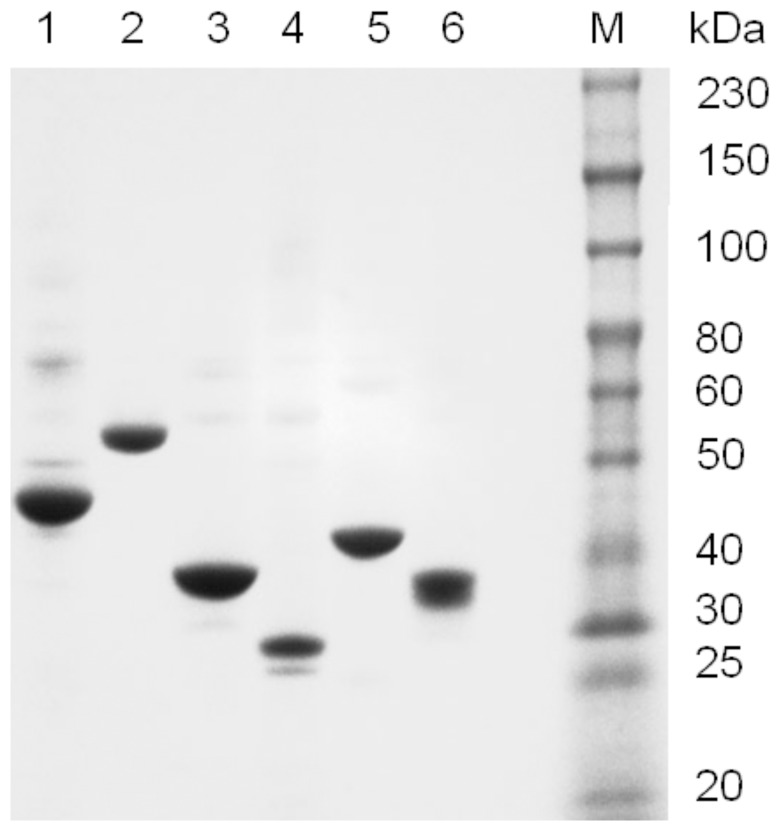
SDS-PAGE of purified methyltransferases. Lane M is the ColorPlus protein ladder (NEB # P7710). Lane 1: M.MspI, Lane 2: M.AluI, Lane 3: M.EcoRI, Lane 4: M.EcoKDam, Lane 5: M.HpaII, and Lane 6: M.HaeIII.

Eight out of a total of the 11 commercially available prokaryotic methyltransferases from New England Biolabs were fused with a cleavable intein and chitin binding domain and purified to homogeneity (Figure 1). The stock concentrations of enzymes used for the fidelity determinations were high in order to detect the star activity and varied based on the expression levels of the proteins. The stock concentrations were as follows: M.AluI 20 mg/mL; M.EcoRI 43 mg/mL; M.HhaI 2.8 mg/mL; M.MspI 17 mg/mL; M.EcoKDam 1.0 mg/mL; M.HpaII 9.5 mg/mL; M.HaeIII 4.3 mg/mL; M.BamHI 7.9 mg/mL.

The CpG methylase, M.SssI and GpC methylase, M.CviPI were eliminated from this analysis because due to the large number of CpG or GpC sites in the genome, complete methylation would require an unreasonably high concentration of protein. In addition, M.*Taq*I exhibited an extremely low specific activity which would also require too high a concentration of protein to determine the FI.

### Restriction enzyme digestion protection assay versus radioactive methylation assays

**Figure 2:**
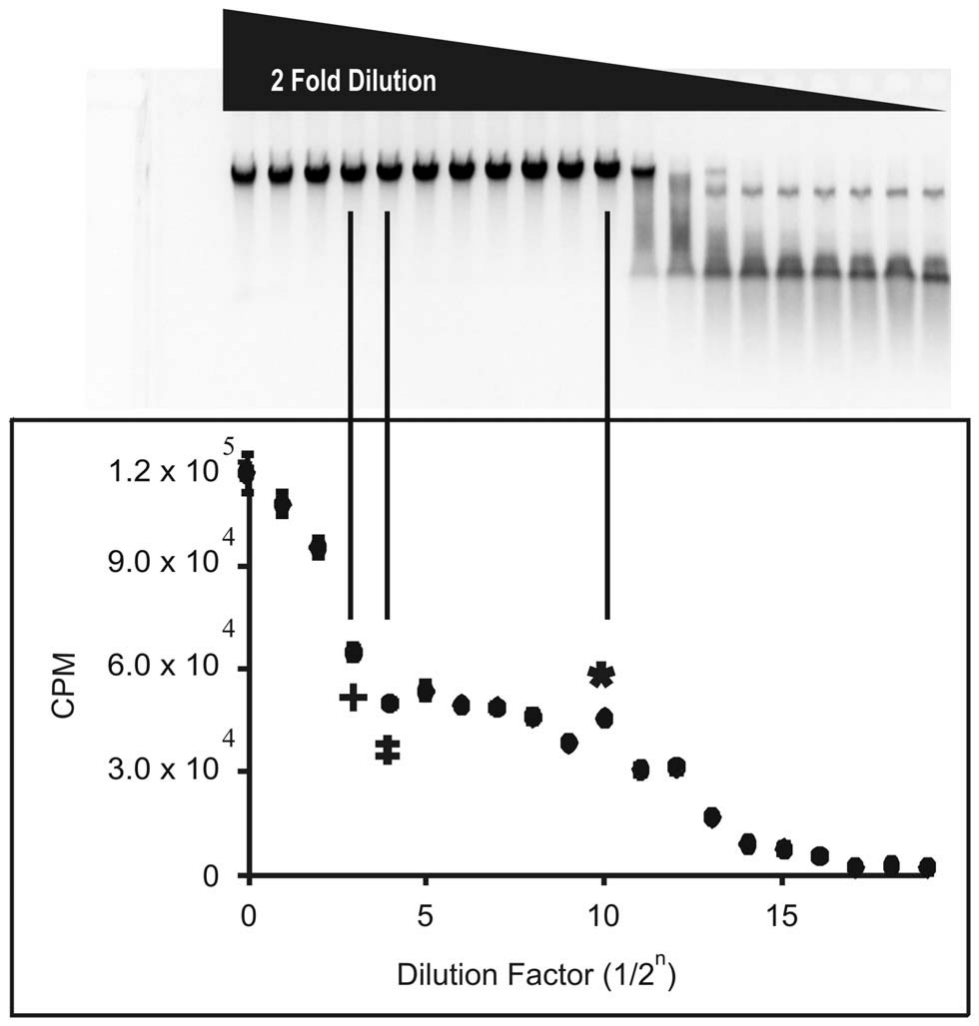
Comparison of a restriction enzyme digestion protection assay and radioactive methylation assay with λ DNA. Both assays were performed using a two-fold dilution series of M.HhaI. The top portion of the figure represents the extent of protection exhibited by M.HhaI against HhaI RE digestion. The bottom portion of the figure shows the amount of H^3^-methyl incorporation by M.HhaI. The X-axis represents the dilution factor of the M.HhaI, where 0 is the highest concentration of enzyme and corresponds to the highest amount of H^3^-methyl incorporation. In contrast, a dilution factor of 19 represents the lowest concentration and enzyme and corresponds to base level H^3^-methyl incorporation. The asterisk designates the LCF, the double dagger designates the HCN, and the plus sign represents the point at which star activity occurs. Upon comparing the same dilution factors from both assays, both can determine the point at which complete methylation of the cognate site occurs. However, there is an apparent increase in H^3^-methyl incorporation after complete methylation of the cognate site at dilution factor 1/2^3^ in the radioactive methylation assay, indicating the presence of star activity, but there is no observable difference on the gel at the same dilution factor in the protection assay.

Restriction enzyme digestion protection assays are the quickest way to determine if methylation of a DNA substrate is complete. To establish if restriction enzyme digestion protection assays are an adequate method to observe methyltransferase star activity, we performed a protection assay and a radioactive methylation assay for M.HhaI on λ DNA. We observed that while the protection assay for M.HhaI was able to provide information on the activity of M.HhaI at its cognate site, it was unable to identify when and if star activity occurs. In contrast, for the radioactive methylation assay, we observe a spike in radioactive counts (methyl incorporation) after the saturation period clearly indicating that additional sites are becoming methylated and is therefore the point at which star activity occurs (Figure 2).

### The fidelity indexes of the methyltransferases

**Figure 3:**
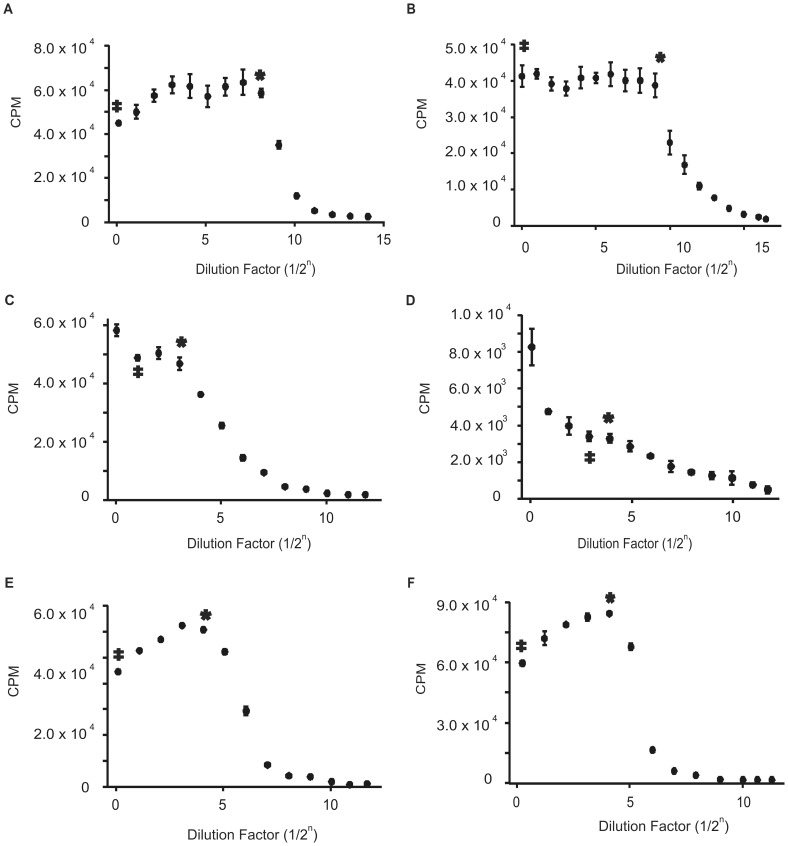
Radioactive methylation assay with *E.* *coli* DNA. On the X-axis, a dilution factor of 0 is the highest concentration of enzyme and corresponds to the highest H^3^-methyl incorporation. Each subsequent data point contains a two-fold decrease in enzyme concentration. The asterisk designates the LCF and the double dagger designates the HCN. FI  =  HCN/LCF. A) M.MspI: At a dilution of 1/2^8^, the substrate is fully methylated and no detectable star activity is observed at the highest enzyme concentration, which is 256-fold over saturation, resulting in an FI of ≥256. B) M.AluI: At a dilution of 1/2^9^, the substrate is completely methylated and no detectable star activity is observed at a dilution of 1/2^0^, which is 500-fold over saturation and results in an FI of ≥500. C) M.HaeIII: Full methylation occurs at a dilution of 1/2^3^, and after a dilution of 1/2^1^, the CPMs start to increase indicating star activity. This results in an FI of 4. D) M.BamHI: At a dilution of 1/2^4^, the substrate is fully methylated and after a dilution of 1/2^3^, the CPMs start to increase indicating star activity, resulting in an FI of 2. E) M.EcoRI and F) M.HpaII: At a dilution of 1/2^4^, the substrate is fully methylated and no detectable star activity is observed at the highest enzyme concentration used, resulting in an FI of ≥16. The slight decrease in CPMs observed in the assay at high concentrations for some enzymes results from the enzymes precipitating out of solution.

**Figure 4:**
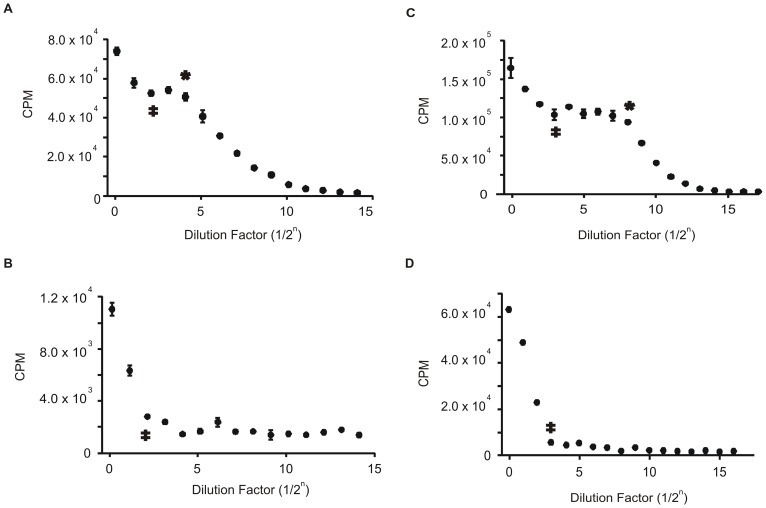
Radioactive methylation assay using *E.* *coli* DNA-comparison between restriction enzyme digested substrate versus undigested substrate. The dilution series and presentation of data are the same as in Figure 3. A) H^3^-methyl incorporation by M.EcoKDam. At a dilution of 1/2^4^, the substrate is fully methylated and after a dilution of 1/2^2^, the CPMs start to increase indicating star activity. This results in an FI of 4. B) H^3^-methyl incorporation by M.EcoKDam with DNA that has been digested with MboI restriction enzyme to remove all M.EcoKDam cognate sites. After a dilution of 1/2^2^, the CPMs start to increase, indicating methylation at non-cognate sites. C) H^3^-methyl incorporation by M.HhaI. At a dilution of 1/2^8^, the substrate is fully methylated and after a dilution of 1/2^3^, the CPMs start to increase indicating star activity. This results in an FI of 32. D) H^3^-methyl incorporation by M.HhaI with DNA that has been digested with HhaI restriction enzyme to remove all M.HhaI cognate sites. After a dilution of 1/2^3^, the CPMs start to increase, indicating methylation at non-cognate sites.

We define the fidelity index (FI) as the ratio of the highest amount of methyltransferase that exhibits no star activity (HCN, double dagger Figure 2, 3, 4) to the lowest amount that exhibits complete modification of the cognate site (LCF, asterisk Figure 2, 3, 4, [8]). While λ DNA was used for the methylation assay comparison, a methyltransferase free strain of *E. coli* was used to determine the FI since it contains approximately 4 million bases and is thus likely to include most of the methylation site variations. We were unable to use *E. coli* for the methylation assay comparison because for the protection assay, the extent of methylation is determined by gel analysis and digestion of *E. coli*'*s* large genome by a restriction enzyme produces a smear instead of a clear digestion pattern. When no star activity was seen even at the highest enzyme concentration assayed, a greater-than or equal sign is given. The FIs for the methyltransferases are listed in Table 1.

**Table 1:** Fidelity Indexes for the Methyltransferases with *E. coli* Genomic DNA.

Enzyme	Specificity	Fidelity Index
M.HhaI	G^C5m^CGC	32
M.AluI	AG^C5m^CT	≥500
M.EcoRI	GA^N6m^ATTC	≥16
M.EcoKDam	G^N6m^ATC	4
M.HpaII	C^C5m^CGG	≥16
M.HaeIII	GG^C5m^CC	4
M.MspI	^C5m^CCGG	≥250
M.BamHI	GGAT^N4m^CC	2

M.HhaI was used as a model system to determine the best conditions to induce star activity. To accomplish this, we varied the reaction time, and the methylase, glycerol and DNA concentrations. It has been shown that the star activity for restriction enzymes is greater at higher glycerol concentrations. However, the results suggest that the star activity for the methyltransferases is mainly concentration dependent (data not shown).

M.AluI exhibited the best FI of ≥500, indicating that M.AluI can be used at a concentration of 500 fold over-saturation without causing star activity (Figure 3B). M.MspI exhibited a FI of ≥250, indicating that a good range of concentrations can be used that will not lead to star activity (Figure 3A). M.HhaI (Figure 4B), M.EcoKDam (Figure 4A), M.HaeIII (Figure 3C) and M.BamHI (Figure 3D) all exhibit very low FI at 32, 4, 4 and 2, respectively and should be used with caution. M.EcoRI (Figure 3E) and M.HpaII (Figure 3F) have non-definitive FIs of ≥16. The inability to exactly determine the FI is a result of the enzymes low specific activity. Therefore, a higher concentration is required to determine if the enzymes have either a low or high fidelity index.

In order to rule out the possibility that the observed star activity was a result of high concentrations of methyltransferase sequestering SAM, we repeated the assay for each enzyme in either the absence of DNA or by using heat inactivated methyltransferase. Additionally, to be sure that excess SAM was not binding to the membrane filters the assay was also repeated in the absence of enzyme. The CPM values from all three control experiments were close to background with minimal variation (∼1000–2000 CPM), indicating that none of these possibilities are contributing to the star activity.

It should be noted that star activity does not only occur under extreme conditions. For example, M.MspI and M.AluI exhibit a very high fidelity even at extremely high concentrations while M.HaeIII and M.BamHI exhibit star activity at concentrations close to those required for complete methylation of the cognate site.

### Radioactive methylation assay with restriction enzyme digested *E. coli* DNA

A radioactive methylation experiment was performed with both M.EcoKDam and M.HhaI where the DNA was digested with MboI and HhaI restriction enzyme, respectively, to remove all cognate sites prior to the addition of the methylase (Figure 4B/D). A spike in CPMs was observed at 0.5 mg/mL for M.EcoKDam (Figure 4B) and 0.7 mg/mL for M.HhaI (Figure 4D). This indicates that the increase in CPMs is the result of methylation at sites outside the cognate site. As a control, this experiment was repeated with M.AluI using AluI restriction digested DNA substrate. Only background CPM values were observed, which is consistent with M.AluI exhibiting high fidelity.

## Discussion

DNA methylation is the most prominent form of epigenetic modification in the cell, which can determine a number of biological functions ranging from protection against restriction enzymes to gene regulation. Biochemical characterization of the sequence specificity for a majority of methyltransferases has yet to be achieved. Of the methyltransferases that have been assayed, many have relatively few off-target effects. However, there have been reports of those that exhibit promiscuity. The importance of DNA methylation and observations of promiscuity encouraged us to determine a quantitative measure of methyltransferase fidelity, thereby providing experimental guidelines for the use of methyltransferases.

Typically, a restriction enzyme digestion protection assay is used to determine the activity of methyltransferases. To verify that methylation is complete, the DNA is subjected to digestion by the restriction endonuclease that corresponds to the methylase. One drawback of this method is that methylation of the DNA on only one strand will protect the DNA against restriction enzyme digestion. Therefore, it is not clear whether or not complete methylation has occurred. Secondly, while this assay will determine if the enzyme has methylated the DNA at its cognate site, it cannot determine if methylation has also occurred at non-cognate sites (Figure 2). In contrast, the degree of methyl incorporation through a radioactive methylation assay is not reliant on restriction enzyme digestion. We believe a saturation period in the radioactive methylation assay indicates complete methylation on both strands since it is known that methyltransferases transfer methyl groups one at a time and then dissociate from the DNA after each catalytic event opposed to linear diffusion, where the enzyme slides across the DNA scanning for unmodified sites [17]. Moreover, we observed an increase in methyl incorporation after the saturation period which indicates that sites other than the cognate site were being methylated. Therefore, to determine the fidelity indexes of the methyltransferases, we used a radioactive methylation assay which enabled us to identify the exact concentration at which star activity occurs.

For most of the methyltransferases we identified a high correlation between the FIs and what is reported in the literature. For example, through SMRT DNA sequencing, which can directly identify methylated DNA bases, *Clark, et al*. determined that M.AluI has a high specificity for its recognition sequence, supporting our reported FI of ≥500 for M.AluI (Figure 3B, [9]). Similarly, the FI of M.MspI of ≥250 (Figure 3A) is supported by *Dubey, et al.* who determined that in the presence of SAM, M.MspI has a 100-fold higher specificity toward its cognate sequence over non-canonical sites [18].

Extremely high concentrations of enzyme are required to properly determine the FI. Therefore a non-definitive FI of ≥16 indicates that the enzyme has a low specific activity. M.EcoRI, which has an FI of ≥16 (Figure 3E), signifying either low or high fidelity, was characterized as a promiscuous methyltransferase by *Berkner, et al.* and *Woodbury, et al.,* leading us to believe that the FI is likely equal to 16 opposed to greater than 16 [12], [14]. In addition, *Berkner and Woodbury* also determined that under standard assay conditions, M.EcoRI methylation of non-cognate sites can only be accomplished by increasing the enzyme concentration, which supports our finding that star activity of the methyltransferases is concentration dependent. In contrast to M.EcoRI, M.HpaII was classified as having high specificity by *Clark, et al.,* suggesting its FI of ≥16 (Figure 3F) is likely greater rather than equal to 16 [9]. Furthermore, the poor FI of M.HaeIII (Figure 3C) is supported by *Cohen, et al.* who determined that it can methylate cytosines in a variety of contexts [10] and the poor FI of M.EcoKDam (Figure 4A) is supported by *Clark, et al.* who identified that it can methylate sites differing only by one base from its recognition sequence [9]. Lastly, through a kinetic characterization, *Youngblood, et al.* determined that M.HhaI has both higher affinity and catalytic efficiency toward its cognate site over its non-cognate site, which contradicts our lower FI of 32 (Figure 4B, [13]). However, the highest concentration used in their kinetic assay is much lower than that used in our assay which is likely to account for the discrepancy. Moreover, we observed a difference in the FI of M.HhaI when using *E. coli* (FI = 32) versus **λ** DNA (FI = 64, Figure 2), which is likely the result of a different ratio of star sites to cognate sites within different DNA substrates. This is supported by *Wei, et al.,* who observed a variance in FI with NotI restriction enzyme when using different DNA substrates, which was attributed to the lack of star sites in one of the substrates, thereby creating a very different star site to cognate site ratio between the two substrates [8].

The extremely low FI of M.BamHI (Figure 3D) is supported by *Nardone, et al.,* who observed hypermethylation on a variety of DNA substrates [19]. It should be noted that although *Nardone, et al.* was using extreme assay conditions to determine if M.BamHI would exhibit relaxed specificity (high glycerol concentrations, pH and ionic strength-conditions known to promote relaxed specificity in restriction enzymes) and our buffer conditions are relatively standard (based on NEB protocols), the conclusions were the same. Furthermore, the reported literatures which support all of our observed FIs use different buffer conditions than those used in our experiments. While we cannot rule out the possibility that the FIs of some methyltransferases may be altered under different assay conditions, we believe that the FIs of the reported enzymes will not change under a variety of conditions.

M.EcoKDam and M.HhaI were used to further validate the observation of star activity. Figure 4 compares the methylation of two *E. coli* DNA substrates: DNA that has been pre-digested with restriction enzyme prior to methylation in order to digest all cognate sites (Figure 4B/D), and DNA that has all sites intact (Figure 4A/C). The results show a spike in methyl incorporation for the undigested substrate that occurs at the same concentration as with the pre-digested substrate for both M.EcoKDam (Figure 4A/B) and M.HhaI (Figure 4C/D). This confirms that methylation is occurring at sites other than the cognate site.

Moreover, our results suggest that enzymes will not simply exhibit star activity when present in excess concentrations over the concentration of the cognate sites. With nearly all enzymes assayed, the point of complete methylation of the cognate sites is followed by a flat region of constant CPMs even though the enzyme concentration is increasing with every data point. The enzymes that exhibit star activity only do so at high enzyme concentrations after the flat region of constant CPMs (Figure 3). Additionally, in the assay where the DNA was pre-digested with restriction enzyme to remove all cognate sites, a spike in methyl incorporation would be observed at all enzyme concentrations if star activity can occur when the enzyme is in excess of the cognate sites (in this case zero). However, we only see a spike in methyl incorporation at high enzyme concentrations (Figure 4B/D) and constant background CPM values in our control with M.AluI (data not shown).

Different degrees of fidelity are required for DNA methyltransferases depending on the intended application. For example, in vitro protection against the paired RE will not be affected if the methyltransferase exhibits star activity. Alternatively, there have been reports where promiscuous functions were beneficial in engineering enzymes with novel specificities. For example, *Rockah-Shmuel, et al.*, used native M.HaeIII promiscuous sites as the first targets for directed evolution studies which resulted in variants of M.HaeIII that methylated novel target sequences and lost specificity toward the original target site [20]. Furthermore, *Cohen, et al*. used the ability of WT M.HaeIII to methylate promiscuous sites as initial selection criteria for the development of a laboratory-evolved M.HaeIII variant that methylates new target sites more efficiently than WT M.HaeIII methylates the canonical site [21]. In contrast, the fidelity of the methyltransferase is critical when DNA is subject to digestion by certain methylation specific enzymes, or when methylation at a precise site will affect gene function. The fidelity indexes will provide a useful guide for methyltransferase applications by the scientific community.
